# Two subtypes of compulsive sexual behavior disorder

**DOI:** 10.3389/fpsyt.2023.1248900

**Published:** 2023-11-09

**Authors:** Sarah Golder, Charlotte Markert, Rhea Psarros, Julian Peter Discher, Bertram Walter, Rudolf Stark

**Affiliations:** ^1^Department for Psychotherapy and Systems Neuroscience, University of Giessen, Giessen, Germany; ^2^Bender Institute of Neuroimaging, University of Giessen, Giessen, Germany; ^3^Center for Mind, Brain and Behavior (CMBB), Phillips-University Marburg and Justus-Liebig-University Giessen, Marburg/Giessen, Germany

**Keywords:** behavioral addiction, cluster analysis, compulsive sexual behavior, subtypes, reinforcement

## Abstract

Models explaining addictive behaviors such as the Interaction of Person-Affect-Cognition-Execution (I-PACE) model emphasize the importance of reinforcement mechanisms for developing and maintaining these behaviors, including compulsive sexual behavior disorder (CSBD) as well as personal characteristics as vulnerability factors. This study aimed to determine whether there are CSBD subtypes distinguished by reinforcement sensitivity. We hypothesize that one subtype is sensitive to positive reinforcement (C^+^subtype) and one is sensitive to negative reinforcement (Ȼ^−^subtype). We calculated a cluster analysis with data from 62 patients with CSBD and tested differences between the identified clusters by *t*-test. The sample consisted only of men. Cluster variables were: the sensitivity to the *Behavioral Inhibition* and *Approach System* (BIS/BAS), the severity of depressive symptoms (BDI-II), the severity of *Trait Anxiety* (STAI-T), *Sexual Sensation Seeking* (SSSS), *Thrill- and Adventure-Seeking* (SSS-V subscale), *Disinhibition* (SSS-V subscale), *Experience Seeking* (SSS-V subscale), and *Boredom Susceptibility* (SSS-V subscale). Between-cluster differences were analyzed for *Trait Sexual Motivation* (TSMQ) and *Sexual Compulsivity* (SCS). The results showed a two-cluster solution with cluster 1 representing patients sensitive to negative reinforcement (Ȼ^−^subtype) and cluster 2 representing patients sensitive to positive reinforcement (C^+^subtype). No significant difference in symptom severity of *Sexual Compulsivity* between clusters was found. Cluster 2 showed higher *Importance of Sex* and a higher motivation to seek sexual encounters than cluster 2. We found a two-cluster solution regarding reinforcement sensitivity in patients with CSBD. This may have clinical implications regarding individual therapy by focusing on the underlying maintenance mechanisms.

## 1. Introduction

In recent decades, out-of-control sexual behavior, commonly known as sexual addiction, hypersexuality, or compulsive sexual behavior (CSB) has become a growing topic of scientific and societal attention (see, e.g., 1–8). It can include or combine, e. g., masturbation, excessive dating, cybersex (e. g., sex chats), telephone sex, and visiting prostitutes excessively (9–11) and the most common form of CSB pornography use (12). The estimated prevalence is about 2% to 9.6%, with men (between 3% and 17.2%) being affected more often than women [between 1.2% and 7.9%; (13–20)]. However, due to the lack of classification in the 10th revision of the International Classification of Diseases [ICD-10; (21)], many researchers and practitioners have used different conceptualizations of clinically relevant CSB (2, 5). On study using gender-specific measurements to assess online sex addiction for example found a higher prevalence in women at 22.9% than in men at 5%. Furthermore, only a few studies estimated the prevalence in a general sample. Therefore, prevalences so far should be interpreted with caution (22).

Before the diagnosis of compulsive sexual behavior disorder (CSBD) was included in the latest version of the International Statistical Classification of Diseases and Related Health Problems [ICD-11; (23)] one often used conceptualization of clinically relevant CSB has been Kafka’s (9) criteria for hypersexual disorder, which are quite similar to CSBD. According to Kafka (9), hypersexual disorder is characterized by repeating sexual fantasies, urges, or behaviors. The ICD-11 characterizes CSBD as repeating out-of-control sexual behavior due to intense sexual impulses or urges (23). Thus, the main difference of the main criterion lies in the specification of the ICD-11 that repeating sexual urges or impulses leads to repetitive out-of-control sexual behavior that is missing in the definition of hypersexual disorder. Regarding other primary criteria of the disorders both disorders include a time criterion. While this time criterion is clearly determined in the diagnostic criteria for hypersexual disorder with at least six months (9) the time criterion for CSBD is rather a proposal of, e.g., six months (23). However, it can be said that the time criterion is nearly the same. Additionally, both diagnoses define that out-of-control sexual behavior must result in salient distress and impairment in several areas of life or functioning (9, 23). Thus, in essence, distress and impairment caused by the problem is a prerequisite in both disorders, but in hypersexual disorder, the problem consists not only of behavior but also of thoughts and the inner feeling of an urge (9), and it does not consider whether this arises only from moral judgments as an exclusion criterion like the diagnoses of CSBD does (23). For both disorders, the problem may not be due to substance use or medication (9, 23). Furthermore, when diagnosing CSBD, the problem should not be better explained by other mental or medical disorders (23). As already in relation to the distress criterion there are also differences between both disorders regarding those differential exclusion criteria. Therefore, those criteria and the distress and impairment criterion of hypersexual disorder cannot be applied one-to-one to those of CSBD. In contrast the differential exclusion criteria of paraphilic disorders is a criterion in hypersexual disorder and CSBD. For both disorders, a comorbid diagnosis of paraphilic disorders is possible (9, 23). Regarding the secondary criteria, at least three of five must be met for hypersexual disorder and at least one of four must be met for CSBD. The main difference in the secondary criteria is that hypersexual disorder includes the criteria of mood modification and coping (9) which is only an additional clinical feature but no secondary criteria in CSBD (23). Additionally, contrary to hypersexual disorder CSBD includes the criteria of dissatisfaction (9, 23). Again, there are several overlaps, but it’s not one-to-one the same. However, both diagnoses share core criteria, and we think that the hypersexual disorder criteria are the closest precursor to capturing the disorder of CSB before the release of ICD-11.

The recently published ICD-11 (23) characterizes CSBD as repeating out-of-control sexual behavior or intense sexual impulses or urges. This behavior becomes a central part of the person’s life and negatively affects one’s health, body care, and familial, social, or educational areas of life. Affected people repeatedly tried to reduce that sexual behavior without success. They are not able to quit, despite negative consequences or decreasing satisfaction from that sexual behavior. To diagnose CSBD, out-of-control sexual behavior must have existed for a long time, e.g., six months or more, and has caused salient distress and impairment in several areas of life or functioning. Paraphilic disorders (such as voyeuristic disorder or pedophilic disorder) are exclusion criteria only if the individual can exercise some degree of control over their arousal patterns. However, if both the diagnostic criteria for a paraphilic disorder and compulsive sexual behavior disorder are fulfilled, both diagnoses may be given. A further exclusion criterion is when, and if the distress only occurs due to moral judgments and condemnations of sexual impulses, urges, or behaviors (23).

After the inclusion of CSBD in the ICD-11 as impulse control disorder, the debate on the categorization of CSBD continues (see, e.g., 6, 8, 24–27). Some researchers request to categorize CSBD as a behavioral addiction due to several findings indicating similarities between CSBD and other addictive disorders (8, 25). Regarding similarities between CSBD and substance use disorders (SUD), both patients show addiction-related desire thinking (28, 29), craving (30, 31), attentional bias towards addiction-specific stimuli (30, 32), cue-reactivity (33, 34), and show higher activation of the reward system after the presentation of addiction-specific stimuli (35). Additionally, in patients with CSBD and alcohol use disorder, smaller grey matter volumes in the left frontal pole were found compared to control subjects (36). Regarding personality characteristics patients with CSBD as well as SUD have a high expression of neuroticism, impulsivity, and sensation seeking, a low expression of conscientiousness, agreeableness, effortful control, and self-directedness (27). Although several findings show similarities between CSBD and other addictive behaviors, there are still unanswered questions (e.g., methodological evidence that may be questioned regarding the presence of tolerance and withdrawal in CSBD) that should be addressed in future studies before a final decision can be made in this discussion (7). However, if CSBD is understood to be a behavioral addiction, the Interaction of Person-Affect-Cognition-Execution (I-PACE) model, introduced by Brand et al. (37, 38), provides a conceptual framework for understanding the development and maintenance of CSBD. This process model describes the development and maintenance of addictive behaviors as a consequence of interactions (I) between neurobiological and psychological predisposing factors (P), affective (A), and cognitive (C) reactions to situational triggers combined with decreased executive functioning (E) as moderators, and reward expectancies, coping styles, as well as affective and cognitive biases as mediators. Therefore, learned associations will be strengthened through positive reinforcement by seeking rewards and experiencing gratifying effects, and negative reinforcement by avoidance of threats and experiencing compensatory effects (37, 38). Sensitivity to positive reinforcement is consequently associated with approach motivation and sensitivity to negative reinforcement with avoidance motivation (39). Brand et al. (37, 38) postulated two phases of the process: (1) the early stages of the development with a predominance of positive reinforcement; and (2) the later stages of the maintenance with a predominance of negative reinforcement.

Up to date, it is unknown whether some patients with CSBD are more receptive to one of these reinforcement methods. Certainly, differences in sensitivity to the type of reinforcement could reflect different forms of vulnerability to CSBD and could indicate diverse functionality of the behavior, which could have important implications for psychotherapy. In the context of substance abuse, similar approaches have been investigated. Cloninger et al. (40–43) researched this topic extensively, looking at the predisposition to alcoholism. They discovered two types of alcohol addiction vulnerability, each with its own genetic and environmental factors (40–43). The two forms of alcohol abuse prototypes stand for the two ends of a continuous spectrum of manifestation (43). Type I is described by anxiety, depression, and high levels of temperament characteristics *Harm Avoidance*, and *Reward Dependence*, as well as low levels of the temperament characteristic *Novelty-Seeking* (40, 42). Type II is described by *Impulsivity*, high levels of the temperament characteristic *Novelty-Seeking*, and low levels of the temperament characteristics *Harm Avoidance* and *Reward Dependence* (40, 42). Cloninger et al. (44) stated that temperament leads to individual differences in associative conditioning because of its relation to stimulus–response characteristics influencing neuronal systems. *Novelty-Seeking* is related to the *Behavioral Activation System* (44, 45), also called the *Behavioral Approach System* [BAS; (46–48)], which is in charge of coordinating goal-oriented behavior in response to reward and non-punitive signaling cues, linked to approach motivation (46, 47). People with a high expression of *Novelty-Seeking* have strong reactions to new stimuli, cues for a potential reward, or possible alleviation of punishment. As a result of that reaction, they engage in more exploratory activities for a reward and actively avoid monotony and possible punishment (44, 45). *Harm Avoidance* is related to the *Behavioral Inhibition System* [BIS; (44, 45)]. It reacts to potentially dangerous and innate anxiety stimuli (stimuli announcing punishment or lack of reward and certain forms of novelty) that create an approach-avoidance conflict (48). Consequently, it ensures the avoidance of punishment. According to Cloninger (44, 45) persons with a high expression of *Harm Avoidance* have strong reactions to cues of aversive stimuli. Because of that, they learned to inhibit behavior to avoid punishment, newness, and frustration due to a lack of rewards (44, 45). It can be concluded that the temperament facets of *Novelty-Seeking* and *Harm Avoidance* lead to different sensitivity to positive and negative reinforcement. Therefore, type I seems to reflect the type that is sensitive to negative reinforcement, and type II, the type that is sensitive to positive reinforcement (43).

The differentiation of patients concerning their reinforcement sensitivity might also be important in persons with CSBD. Neuroimaging studies have found increased activity in reward-related brain regions during the presentation of sexual stimuli, in healthy men and women (49–52). Additionally, this effect was found to be larger in males with CSB than without CSB (34, 53). Thus, men with CSB appear to be even more sensitive to the reward value of sexual stimuli. Increased activity in reward-related brain areas due to presented sexual stimuli leads to an approach motivation towards such stimuli. In the neurophenomenological model of sexual arousal, by Stoléru et al. (51) reward-related brain areas are involved in the motivational component, which includes the processes that lead behavior toward a sexual goal. This is reflected by increased approach motivation towards visual sexual stimuli (54, 55). Furthermore, Costumero et al. (56) found an association between BAS and reward sensitivity in the neural processing of sexual stimuli in a non-clinical sample. These findings lead to the assumption of a general sensitivity to positive reinforcement regarding sexual stimuli, which is consistent with the previously reported conclusion that sexual stimuli *per se* are rewarding. It indicates the sensitivity to positive reinforcement in patients with CSB. Several studies found that the approach motivation towards sexual stimuli is higher in persons with high severity of problematic sexual behavior (57) and high severity of problematic pornography use (57–59). Additionally, in one study persons with problematic pornography use showed a higher approach motivation toward sexual stimuli than persons without problematic pornography use (59). However, Kahveci et al. (55) did not find a higher approach motivation in persons with higher severity of problematic pornography use or a significant difference between users with and without a problematic consumption pattern. Overall, however, several studies indicate that there is some relationship between BAS and CSBD. Additionally, CSB is associated with *Sensation Seeking* (60) and *Sexual Sensation Seeking* (61, 62). *Sensation Seeking* describes the personality characteristic of searching for stimulation, the tendency to create new and intensive experiences and take risks for them (63), and therefore, it is a trait that reflects a high sensitivity to positive reinforcement.

Bancroft and Vukadinovic (64) found in a comparison of people with and without sex addiction that the group of patients with self-defined sex addiction had a substantial tendency to experience greater sexual interest when they were depressed or anxious. Depression and anxiety are mental disorders often accompanied by avoidance behavior (65–67). Anxiety is equivalent to activation in the BIS (68). In patients with depression and anxiety, a greater likelihood of using maladaptive cognitive emotion regulation strategies was found with an increase in BIS sensitivity (69). Bancroft and Vukadinovic (64) assume that in people with sexual addiction, negative moods associated with heightened arousal (namely anxiety) lead to sexual arousal via excitation transfer and that they try to compensate for their negative mood by experiencing sexual release through orgasm. However, Bancroft and Vukadinovic (64) did not find affect to be significant in all cases of sexual addiction. As a result, their findings suggest that a subset of patients with CSBD is more sensitive to negative reinforcement.

However, such differences in personal reinforcement sensitivity in patients with CSBD are still uncharted. To our knowledge, no study to date has examined potential subtypes of CSBD distinguished by the type of reinforcement. Based on the assumptions of the I-PACE model that both forms of reinforcement sensitivity are involved in the development and maintenance of behavioral addiction, we assume the existence of two subtypes of CSBD, regarding differences in their reinforcement sensitivity. According to Cloninger et al. (43), we anticipate that the prototypes of these two subtypes will stand for the two ways resulting in CSBD depending on person characteristics. Therefore, we expect one subtype to be sensitive to positive reinforcement and use sexual behavior to experience gratifying effects (C^+^subtype) and one subtype to be sensitive to negative reinforcement and use sexual behavior to experience compensatory effects (Ȼ^-^subtype). We hypothesize that (1) the C^+^subtype shows higher values of BAS, *Sensation Seeking*, and *Sexual Sensation Seeking*, and (2) the Ȼ^−^subtype shows higher values of BIS, depression, and anxiety. This study aimed to provide evidence for the existence of two subtypes of patients with CSBD differing in their sensitivity to reinforcement.

## 2. Materials and methods

### 2.1. Participants and procedure

**Figure 1:**
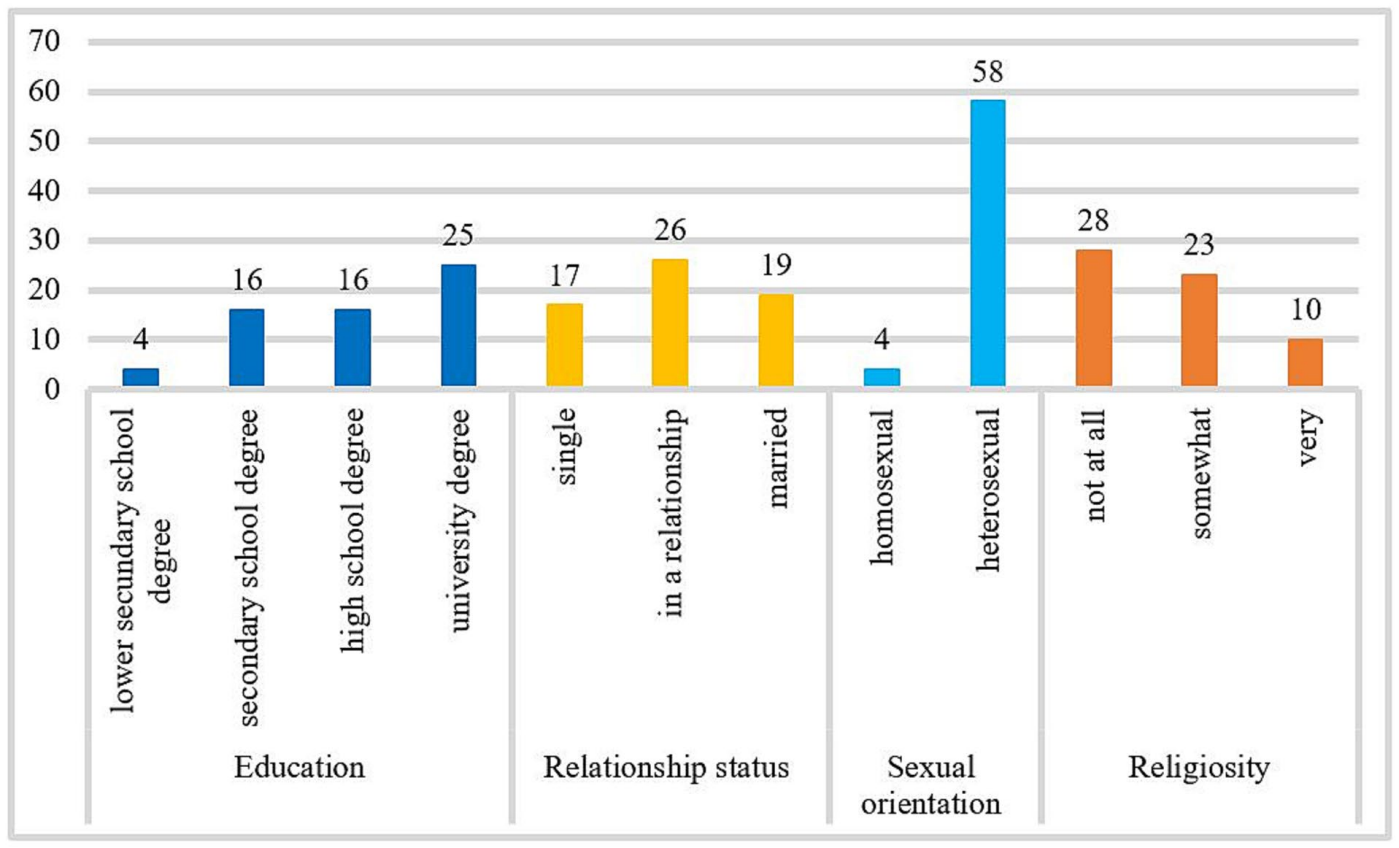
Frequency distribution of the variable education, relationship status, sexual orientation, and religiosity. The number of cases included in the frequency analysis for education and religiosity was *n* = 61, and for relationship status and sexual orientation was *n* = 62.

Data from 62 men aged 20–68 (*M* = 37.21 years, SD = 11.63) seeking treatment for CSBD at the outpatient psychotherapy clinic at the University of Giessen were collected from 2012 to 2021. At the time of participation, patients were not in treatment. Participation was not compensated financially. Three patients were excluded from the study due to a lack of responses. To be included in the cluster analysis, Kafka’s (9) criteria for the hypersexual disorder had to be fulfilled. There were no exclusion criteria. For further description of the sample, see Figure 1.

### 2.2. Diagnosis criteria for CSBD

The criteria of hypersexual disorder outlined by Kafka (9) had to be met to receive a diagnosis of CSBD. According to Kafka (9), people with hypersexual disorder have intense, repetitive sexual fantasies, impulses, or behaviors that fulfill at least three of the following five criteria: (1) time spent has a negative impact on other non-sexual goals, activities, and commitments, (2) it is used as a reaction to negative moods (e. g. anxiety, depression), (3) it is used as a reaction to stressful life events, (4) repeated failures to control or reduce attempts, and (5) repetitive execution despite the risk of physical or emotional injury to oneself or others. These sexual fantasies, impulses, or behaviors must exist for six months or longer, and induce clinically relevant psychological distress or impairment in important areas of life. Additionally, the diagnosis is only assigned, if sexual fantasies, impulses, and behaviors are not caused through exogen substances such as drugs or medications (9). Because the criteria for CSBD were not known in 2012, the hypersexual disorder criteria are the best precursor for capturing CSBD.

### 2.3. Ethical issues

The procedure of data collection was in accordance with the Declaration of Helsinki. The survey was voluntary for all the patients. Receiving treatment was unaffected by the decision to participate or not. All patients gave informed consent about using their data anonymously for research.

### 2.4. Materials

#### 2.4.1. BIS/BAS Scales

The BIS/BAS Scales (70) assess the sensitivity to the BIS with BIS scale (e. g., “I worry about making mistakes”) and the BAS with BAS scale (e. g., “I crave excitement and new sensations”) via two scales. The BIS scale reflects one’s susceptibility to fear based on their reaction to fear-inducing situations (e. g., impending punishment). The BAS scale reflects one’s reward sensitivity and consists of three subscales: *Reward Responsiveness*, *Fun Seeking*, and *Drive*. Participants answered the 24 statements of the questionnaire on a 4-point Likert scale (1 = “very true for me” to 4 = “very false for me”). The internal consistencies of the BIS scale (Cronbach’s α = 0.67) and the BAS scale (Cronbach’s α = 0.82) in the present study were questionable to good. The internal consistencies of the BAS subscales *Reward Responsiveness* (Cronbach’s α = 0.63), *Fun Seeking* (Cronbach’s α = 0.54), and *Drive* (Cronbach’s α = 0.81) were poor to good in the present study. The BIS scale, BAS subscale *Reward Responsiveness*, and BAS subscale *Fun Seeking* with poor to questionable reliability were also included in the analysis.

#### 2.4.2. State–Trait Anxiety Inventory

The trait version of the State–Trait Anxiety Inventory [STAI-T; (71)] assesses anxiety as a trait via 20 items (e. g., “I lack self-confidence”). Respondents rate their agreement on a 4-point Likert scale (1 = “almost never” to 4 = “almost always”). The internal consistency of the Trait Anxiety Scale was excellent in the present study (Cronbach’s α = 0.92).

#### 2.4.3. Beck-Depression-Inventory II

The Beck-Depression-Inventory II [BDI-II; (72)] assesses the severity of depressive symptoms via 21 items (e. g., “sadness”). For each of the 21 symptoms, four statements are given from which the participant selects the statement that best describes how he has felt in the past two weeks. The 4-point Likert scale ranges from 0 to 3. The internal consistency of the BDI-II was excellent in the present study (Cronbach’s α = 0.93).

#### 2.4.4. Sensation Seeking Scale

*Sensation seeking* was assessed with the Sensation Seeking Scale, Form V [SSS-V; (73)], via 40 items. Participants had to choose one of two response options per item. *Sensation Seeking* consists of four subscales: *Thrill- and Adventure-Seeking* (e. g., “I would like to try surf-board riding”), *Disinhibition* (e. g., “I like “wild” uninhibited parties”), *Experience Seeking* (e. g., “I have tried marijuana or would like to”), and *Boredom Susceptibility* (e. g., “I get bored seeing the same old faces”). The internal consistencies of the subscales *Thrill- and Adventure-Seeking* (Cronbach’s α = 0.80), *Disinhibition* (Cronbach’s α = 0.72), *Experience Seeking* (Cronbach’s α = 0.60), and *Boredom Susceptibility* (Cronbach’s α = 0.59) were poor to good in the present study. The scales with poor and questionable reliability were also included in the analysis.

#### 2.4.5. Sexual Sensation Seeking Scale

*Sexual sensation seeking* was assessed with the German version of the Sexual Sensation Seeking Scale [SSSS; (74)] via 11 items (e. g., “I feel like exploring my sexuality”). Participants answered on a 4-point Likert scale (1 = “not at all like me” to 4 = “very much like me”). The internal consistency of the SSSS was good in the present study (Cronbach’s α = 0.80).

#### 2.4.6. Sexual Compulsivity Scale

Symptom severity of sexual compulsive behavior was assessed with the German version of the Sexual Compulsive Scale [SCS; (74)] via 10 items (e. g., “My desires to have sex have disrupted my daily life”). Respondents must rate their agreement on a 4-point Likert scale (1 = “not at all like me” to 4 = “very much like me”). The internal consistency of the SCS was good in the present study (Cronbach’s α = 0.89).

#### 2.4.7. Trait Sexual Motivation Questionnaire

The *Trait Sexual Motivation* was assessed with the Trait Sexual Motivation Questionnaire (TSMQ) from Stark et al. (75) via 35 items. The questionnaire consists of four subscales: *Solitary Sexuality* (e. g., “I masturbate regularly“), *Importance of Sex* (e. g., “Sex is important to me”), *Seeking Sexual Encounters* (e. g., “I often go out to find a partner for sex”), and *Comparison with Others* (e. g., “Most people want less sex than me”). Respondents answer on a 6-point Likert scale (0 = “not at all” to 5 = “very much”). The internal consistencies of the TSMQ total score (Cronbach’s α = 0.94) and subscales *Solitary Sexuality* (Cronbach’s α = 0.85), *Importance of Sex* (Cronbach’s α = 0.91), *Seeking Sexual Encounters* (Cronbach’s α = 0.89), and *Comparison with Others* (Cronbach’s α = 0.92) were good to excellent in the present study.

### 2.5. Statistical analysis

Firstly, missing values were calculated via multiple regression using R 3.5.2 (76). If there were more than 80% missing values per scale, the participant’s data set was excluded from the calculation of missing values for these scales. One subject had more than 20% missing values in the STAI-T scale, one subject in the BDI-II scale, and one subject in each of the SSS-V scales and the BIS/BAS scale. Descriptive analyses were conducted using IBM SPSS Statistics (Version 27).

Secondly, clusters were calculated using R 3.5.2 (76). Z-standardized scores of the BIS/BAS, BDI-II, STAI-T, and SSSS scales, as well as SSS-V subscales (*Thrill- and Adventure-Seeking*, *Disinhibition*, *Experience Seeking*, *Boredom Susceptibility*), were utilized to compute clusters. The number of clusters for all subjects was determined using the gap statistic (77). Clusters were calculated for BIS/BAS, STAI-T, BDI-II, and SSSS scales as well as SSS-V subscales via k-medoids clustering (78).

Finally, differences between clusters were analyzed via *t*-test using IBM SPSS Statistics (Version 27) with z-standardized scores of BIS/BAS, BDI-II, STAI-T, SSSS, SSS-V subscales, SCS, and TSMQ total score as well as subscales. The statistical significance was determined using a *p* < 0.05 significance level.

## 3. Results

### 3.1. Participants

**Figure 2:**
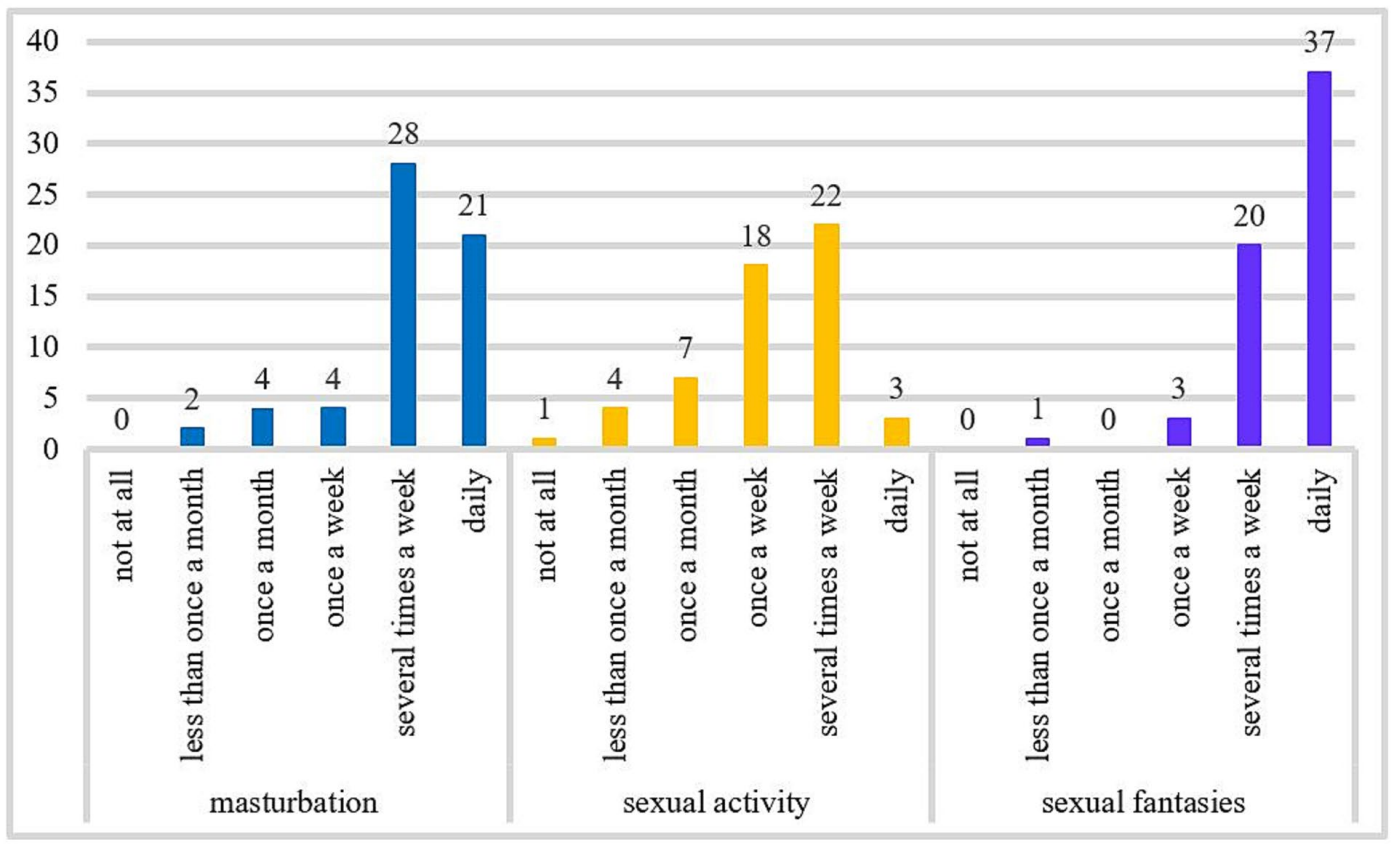
The frequency of masturbation, sexual activity, and sexual fantasies per month. The number of cases included in the frequency analysis for monthly masturbation was *n* = 59, for the frequency of sexual activity was *n* = 55, and for sexual fantasies was *n* = 61.

This sample is mostly made up of employed (73.8%), and childless people (62.9%) who have had sexual relationships in the last five years (87.1%). Almost all patients had experience with pornography (96.8%). Pornography is consumed on average *M* = 30.17 h (SD = 33.48; *n* = 26) per month. Most patients reported problems with their pornography use patterns (90.3%), some patients reported problems with prostitution (13.1%), and some with excessive dating (11.5%). For further information about the samples’ sexual activity, see Figure 2.

On average, the sample shows clinically relevant *Trait Anxiety* (STAI-T), mild severity of depressive symptoms (BDI-II), moderate to high symptom severity of compulsive sexual behavior (SCS), moderate need for sexual intensity and diversity (SSSS), high sensitivity of BIS and BAS, a moderate tendency to engage in sports and other activities that involve danger or speed (SSS-V *Thrill- and Adventure-Seeking*), towards socially and sexually uninhibited behavior (SSS-V *Disinhibition*), to search for experiences through unconventional lifestyle and travel (SSS-V *Experience Seeking*), a moderate aversion to repetition and routine (SSS-V *Boredom Susceptibility*), a moderate overall *Trait Sexual Motivation* (TSMQ), a high *Trait Sexual Motivation* for relationship independent sexual activities (TSMQ *Solitary Sexuality*), a moderate to high motivation for sexual arousal and activity (TSMQ *Importance of Sex*), a low to moderate motivation to get in touch with new sexual partners (TSMQ *Seeking Sexual Encounters*), and a moderate to high self-perceived sexual motivation in comparison to other persons (TSMQ *Comparison with Others*). For statistical information, see Table 1.

**Table 1:** Mean scores and standard deviations (in parenthesis) for all questionnaires and subscales.

	*M* (SD)
BAS [1, 4]^1^	3.01 (0.42)
BIS [1, 4]^1^	3.04 (0.47)
STAI-T [1, 4]^2^	2.38 (0.51)
BDI-II [0, 3]^3^	0.82 (0.55)
SSSS [1, 4]^4^	2.86 (0.52)
SSS-V [0, 1]^5^	
*Thrill- and Adventure-Seeking*	0.57 (0.29)
*Disinhibition*	0.48 (0.26)
*Experience Seeking*	0.60 (0.20)
*Boredom Susceptibility*	0.34 (0.21)
SCS [1, 4]^6^	2.84 (0.75)
TSMQ Total [0, 5]^7^	3.17 (0.89)
*Solitary Sexuality* [0, 5]^7^	4.01 (0.84)
*Importance of Sex* [0, 5]^7^	3.79 (0.89)
*Seeking Sexual Encounters* [0, 5]^7^	1.56 (1.50)
*Comparison with Others* [0, 5]^7^	3.33 (1.32)

BAS, Behavioral Approach System; BIS, Behavioral Inhibition System; BDI-II, Beck-Depression-Inventory II; STAI-T, trait version of the State–Trait Anxiety Inventory; SSSS, Sexual Sensation Seeking Scale; SSS-V, Sensation Seeking Scale, form V; SCS, Sexual Compulsivity Scale; TSMQ, Trait Sexual Motivation Questionnaire. ^1^ BIS/BAS: 1 = high agreement – 4 = disagreement. ^2^ STAI-T: 1 = low symptom severity – 4 = high symptom severity. ^3^ BDI-II: 1 = low symptom severity – 3 = high symptom severity. ^4^ SSSS: 1 = no concordance – 4 = high concordance. ^5^ SSS-V: 0 = no sensation seeking – 1 = high sensation seeking. ^6^ SCS: 1 = not at all like me – 4 = very much like me. ^7^ TSMQ: 0 = not at all – 5 = very much.

### 3.2. Cluster analysis

**Figure 3:**
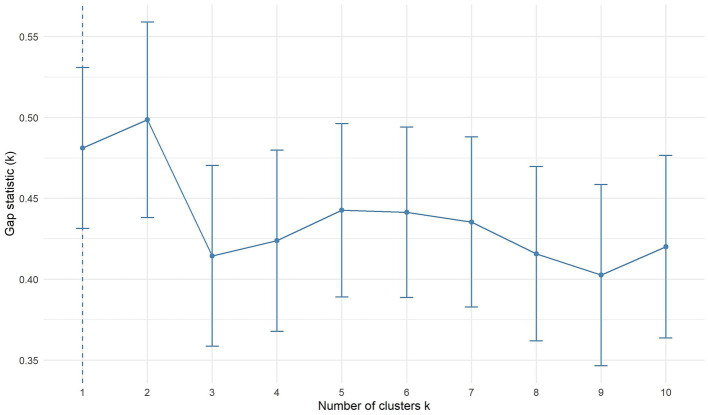
Gap function for BIS/BAS, BDI-II, STAI-T, SSSS, and SSS-V subscales scores. The optimal number of clusters is represented by the highest value. Bars represent ±1 standard error. BAS, Behavioral Approach System; BIS, Behavioral Inhibition System; BDI-II, Beck-Depression-Inventory II; STAI-T, trait version of the State–Trait Anxiety Inventory; SSSS, Sexual Sensation Seeking Scale; SSS-V, Sensation Seeking Scale, form V.

**Figure 4:**
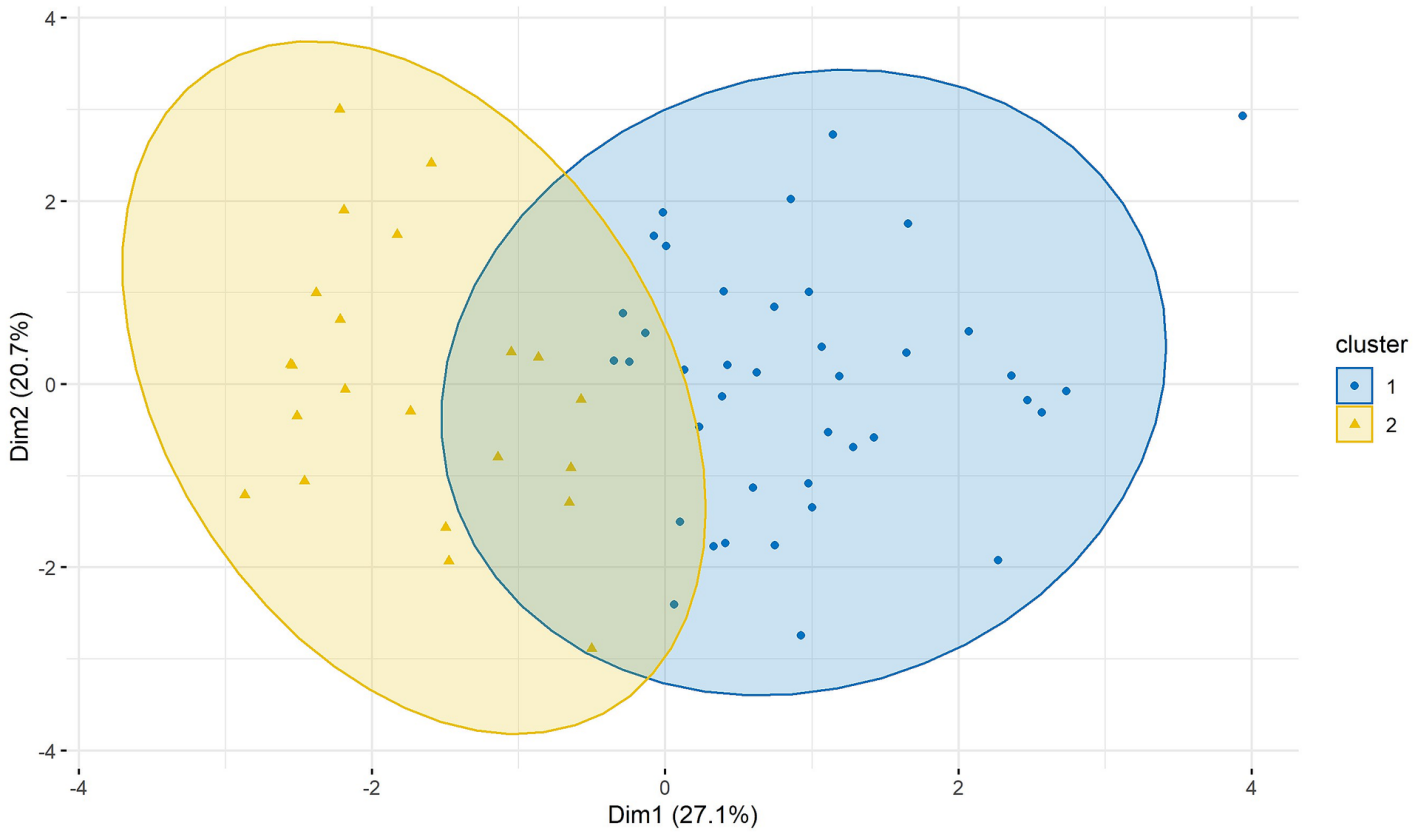
Clusters of BIS/BAS, BDI-II, STAI-T, SSSS, and SSS-V subscales scores in two dimensions (Dim1 and Dim2). Cluster 1 *n* = 40; cluster 2 *n* = 22. BAS, Behavioral Approach System; BIS, Behavioral Inhibition System; BDI-II, Beck-Depression-Inventory II; STAI-T, trait version of the State–Trait Anxiety Inventory; SSSS, Sexual Sensation Seeking Scale; SSS-V, Sensation Seeking Scale, form V.

A two-cluster solution had the highest gap statistic (Figure 3). With k-medoids, we have formed two clusters. 40 patients were assigned to the first cluster and 22 to the second cluster (Figure 4).

#### 3.2.1. Differences Between Clusters

**Figure 5:**
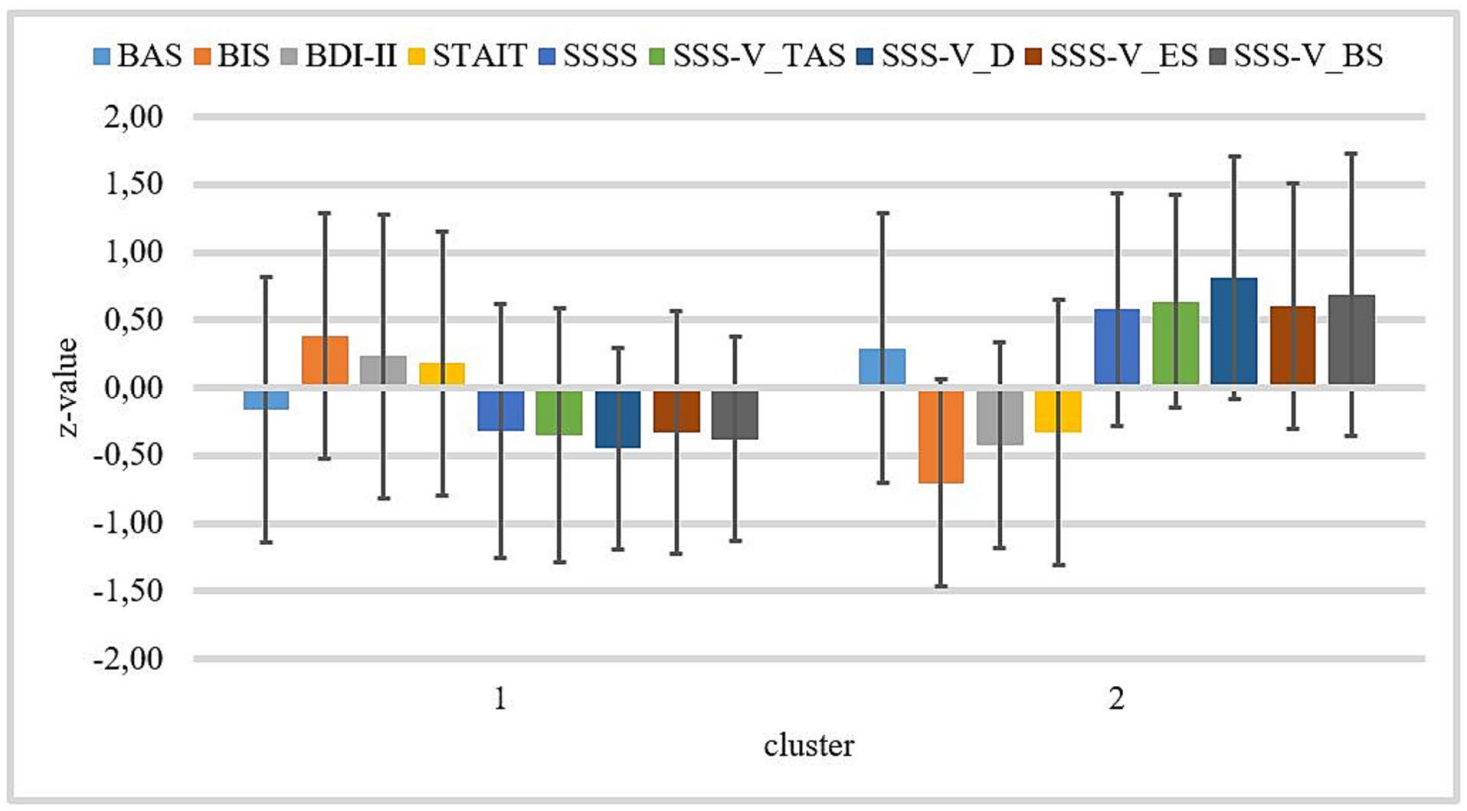
Z-standardized means by both clusters for BIS/BAS, BDI-II, STAI-T, SSSS, and SSS-V subscales. Cluster 1 *n* = 40; cluster 2 *n* = 22. Bars represent standard deviations of the z-standardized means. BAS, Behavioral Approach System; BIS, Behavioral Inhibition System; BDI-II, Beck-Depression-Inventory II; STAI-T, trait version of the State–Trait Anxiety Inventory; SSSS, Sexual Sensation Seeking Scale; SSS-V_TAS, Sensation Seeking Scale, form V, subscale *Thrill- and Adventure-Seeking*; SSS-V_D, Sensation Seeking Scale, form V, subscale disinhibition; SSS-V_ES, Sensation Seeking Scale, Form V, subscale Experience Seeking; and SSS-V_BS, Sensation Seeking Scale, form V, subscale Boredom Susceptibility.

We found no significant difference for BAS and STAI-T between clusters 1 and 2. Patients of cluster 1 have significantly higher BIS and BDI-II values than patients of cluster 2. Patients of cluster 2 show significantly higher values on SSSS, SSS-V subscale *Thrill- and Adventure-Seeking*, SSS-V subscale *Disinhibition*, SSS-V subscale *Experience Seeking*, and SSS-V subscale *Boredom Susceptibility* than patients of cluster 1 (see Table 2). Standardized means of all variables by clusters are visualized in Figure 5.

**Table 2:** Means, standard deviations (in parenthesis), and statistical differences between both clusters.

	Cluster 1	Cluster 2		
	*M* (SD)	*M* (SD)	*t*(60)	*p*
BAS	2.94 (0.41)	3.13 (0.42)	−1.74	0.086
*Reward Responsiveness*	3.24 (0.42)	3.25 (0.50)	−0.08	0.468
*Fun Seeking*	2.81 (0.49)	3.26 (0.37)	−3.70***	< 0.001
*Drive*	2.72 (0.56)	2.85 (0.70)	−0.78	0.219
BIS	3.23 (0.42)	2.71 (0.36)	4.78***	< 0.001
STAI-T	2.47 (0.50)	2.21 (0.50)	1.98	0.052
BDI-II	0.95 (0.58)	0.58 (0.42)	2.60*	0.012
SSSS	2.70 (0.48)	3.16 (0.45)	−3.70***	< 0.001
SSS-V				
*Thrill- and Adventure-Seeking*	0.47 (0.27)	0.76 (0.23)	−4.21***	< 0.001
*Disinhibition*	0.37 (0.19)	0.69 (0.23)	−5.91***	< 0.001
*Experience Seeking*	0.53 (0.18)	0.72 (0.18)	−3.92***	< 0.001
*Boredom Susceptibility*	0.26 (0.16)	0.48 (0.22)	−4.64***	< 0.001

Cluster 1 *n* = 40; cluster 2 *n* = 22. BAS, Behavioral Approach System; BIS, Behavioral Inhibition System; BDI-II, Beck-Depression-Inventory II; STAI-T, trait version of the State–Trait Anxiety Inventory; SSSS, Sexual Sensation Seeking Scale; SSS-V, Sensation Seeking Scale, form V. BAS Scales Reward Responsiveness, Fun Seeking, and Drive: df = 59. * *p* ≤ 0.05. ** *p* ≤ 0.01. *** *p* ≤ 0.001.

For symptom severity, we did not find a significant difference between cluster 1 (*M_1_* = 2.78, SD*_1_* = 0.77) and cluster 2 (*M_2_* = 2.93, SD*_2_* = 0.72; *t*(58) = −0.749, *p* = 0.457). Regarding *Trait Sexual Motivation* clusters differ significantly in TSMQ total score (*M_1_* = 2.90, SD*_1_* = 0.79; *M_2_* = 3.67, SD*_2_* = 0.86; *t*(60) = −3.577, *p* < 0.001), TSMQ subscale *Importance of Sex* (*M_1_* = 3.61, SD*_1_* = 0.87; *M_2_* = 4.12, SD*_2_* = 0.85; *t*(60) = −2.189, *p* = 0.033), and TSMQ subscale *Seeking Sexual Encounters* (*M_1_* = 0.91, SD*_1_* = 1.14; *M_2_* = 2.76, SD*_2_* = 1.35; *t*(60) = −5.750, *p* < 0.001). There were no significant differences in TSMQ subscales *Solitary Sexuality* (*M_1_* = 3.93, SD*_1_* = 0.70; *M_2_* = 4.15, SD*_2_* = 1.05; *t*(60) = −0.954, *p* = 0.344) and *Comparison with Others* (*M_1_* = 3.15, SD*_1_* = 1.40; *M_2_* = 3.67, SD*_2_* = 1.11; *t*(60) = −1.502, *p* = 0.138) between clusters.

## 4. Discussion

This study aimed to provide evidence for the existence of two subtypes of patients suffering from CSBD that differ in their sensitivity to reinforcement. As expected, we found a two-cluster solution to be the optimal number of clusters. Cluster 1 includes patients with higher values in characteristics reflecting a sensitivity to negative reinforcement including increased general activation of the *Behavioral Inhibition System* and more severe depressive symptoms, and cluster 2 includes patients with higher values in characteristics reflecting a sensitivity to positive reinforcement including increased *Sexual Sensation Seeking*, *Thrill- and Adventure-Seeking*, *Disinhibition*, *Experience Seeking,* and *Boredom Susceptibility*.

We found that patients of cluster 1 represent the subtype who is sensitive to negative reinforcement. Consistent with our assumptions, patients of cluster 1 are significantly more sensitive to *the Behavioral Inhibition System*, which is, according to Gray and McNaughton (48), activated by approach-avoidance conflicts through stimuli announcing punishment, lack of reward, or certain forms of novelty to avoid potential danger and to enable potential rewarding experiences. Additionally, Sun et al. (69) found that there is a greater likelihood of using maladaptive cognitive emotion regulation strategies with an increase in *Behavioral Inhibition System* sensitivity in patients with depression and anxiety. Based on the definition of the *Behavioral Inhibition System* (48) and the results of Sun et al. (69), the *Behavioral Inhibition System* is related to coping-motivated behaviors and thus to negative reinforcement. Additionally, patients of cluster 1 showed significantly more symptoms of depression than patients of cluster 2. Symptoms of depression are associated with avoidance behavior (65, 66) and are in line with the conclusion of Bancroft and Vukadinovic (64) that some patients with sexual addiction compensate for their depressive or anxious mood with orgasm. This assumption can be supported by the finding of increased libido as an atypical symptom of depression (79). Our findings indicate a higher sensitivity to negative reinforcement in patients of cluster 1. However, regarding anxiety symptoms, patients of cluster 1 do not significantly differ from patients of cluster 2. This is surprising because Gray (68) equated activation of the *Behavioral Inhibition System* with anxiety. Therefore, we expected that significant differences in the sensitivity of the *Behavioral Inhibition System* led to significant differences in symptoms of anxiety. This lack of significant difference may indicate that both clusters are not the extremes of a continuum as assumed or it could be due to our small sample size. Nevertheless, the significantly higher levels of *Behavioral Inhibition System* sensitivity and depressive symptoms in patients of cluster 1 suggest that patients from this cluster use sexual behavior for compensation. Therefore, despite the lack of significant differences in *Trait Anxiety* between clusters, we conclude that cluster 1 represents the Ȼ^−^subtype.

We found that patients of cluster 2 represent the subtype sensitive to positive reinforcement. As assumed, patients of cluster 2 show significantly higher values of all *Sensation Seeking* dimensions and *Sexual Sensation Seeking* than patients of cluster 1. According to these scales, they have a higher tendency to engage in dangerous or fast activities, uninhibited sexual and social behavior, be more active in searching for experiences, have a higher aversion to repetition and routine (73), and a higher tendency to try various sexual practices, including more frequent unprotected sexual intercourse and more frequently changing sexual partners (61). Considering these characteristics [searching for stimulation, creating new and intensive experiences, and taking risks; (63)], these results suggest that patients in the second cluster have a stronger sensitivity to positive reinforcement than those in cluster 1. However, they do not show the assumed significantly higher sensitivity to the *Behavioral Approach System*. The whole sample showed high sensitivity to *Behavioral Approach System*. Contrary to our assumptions, the sensitivity to *Behavioral Approach System* does not seem to be a good indicator to distinguish between patients with a higher sensitivity to positive reinforcement and those with a higher sensitivity to negative reinforcement. One reason might be that compared to people without hypersexuality, people with hypersexuality score significantly higher in the *Behavioral Approach System* dimension *Fun Seeking* only, not in the other dimensions of the *Behavioral Approach System* (62). The use of the BAS total score may therefore lead to an inaccurate result. Hence, we have subsequently evaluated differences in BAS subscales scores and found patients of cluster 2 to show significantly higher values in the *Behavioral Approach System* dimension *Fun Seeking* compared to patients of cluster 1. Therefore, we conclude that cluster 2 represents the C^+^subtype.

However, all dimensions of the *Behavioral Approach System* reflect a person’s reward sensitivity (70) and therefore, we had expected significant differences between both clusters. One explanation that there are no differences between clusters in the *Behavioral Approach System* dimension *Drive* and the *Behavioral Approach System* dimension *Reward Responsiveness* might be that the increased activity in reward-related brain regions during the presentation of sexual stimuli in patients with CSBD (34, 53) leads to an overall higher activation of the *Behavioral Approach System* and, therefore, to an overall higher approach motivation towards sexual stimuli (51, 54), whether the patient with CSBD has a higher sensitivity to positive or negative reinforcement. This fits with the theory of Gray and McNaughton (48) that the *Behavioral Inhibition System* is activated in approach-avoidance conflicts when a stimulus activates both the *Behavioral Approach System* and the *Fight–Flight–Freezing System* (FFFS). Additionally, it can explain why patients with sex addiction have more sexual interest when in aversive emotional states and, as a result, are more likely to turn to sexual stimulation than healthy men in whom the opposite effect can be observed (64).

If we look at the I-PACE model (37, 38) with regard to the development of CSBD, we can say that the patients of our clusters differ with regard to the interaction of the variables P (predisposing factors), A (affective reaction), and C (cognitive reaction), as well of the mediator reward expectancies and coping style. For example, patients of the Ȼ^−^subtype have a higher sensitivity to the Behavioral Inhibition System as a neurobiological predisposing factor. Additionally, patients of the Ȼ^−^subtype and patients of the C^+^subtype will differ in those factors during the maintenance of CSBD since we can assume that this trait sensitivity to reinforcement remains reasonably stable within individuals. Since the I-PACE model assumes that state sensitivity of reinforcement changes over time, switching from experiencing primary positive reinforcement to experiencing primarily negative reinforcement at later stages (37, 38), it would be exciting to examine this interaction between state and trait sensitivity to reinforcement over a longer period of time in future research. We assumed that patients with CSBD of the C^+^subtype and Ȼ^−^subtype are similar to Cloninger’s patients with alcohol addiction of type I and type II (40–43). Whereas the C^+^subtype reflects patients who are sensitive to positive reinforcement like Cloninger’s type II cluster, and the Ȼ^−^subtype reflects patients who are sensitive to negative reinforcement like Cloninger’s type I cluster (43). Our findings support this assumption. We found increased general activation of the *Behavioral Inhibition System* and more severe depressive symptoms in patients with CSBD of the Ȼ^−^subtype. Patients with alcohol addiction of type I also show more severe depressive symptoms and a higher level of temperament characteristic *Harm Avoidance* (40, 42) that is related to the *Behavioral Inhibition System* (44, 45). It can be concluded that patients with CSBD of the Ȼ^−^subtype and patients with alcohol addiction of type I show characteristics that reflect a higher sensitivity to negative reinforcement. However, contrary to patients with CSBD of the Ȼ^−^subtype patients with alcohol addiction of type I also show a higher symptom severity of anxiety and lower levels of temperament characteristic *Novelty-Seeking* (40, 42) that is related to the *Behavioral Approach System* (44–48) which both reflect the sensitivity to positive reinforcement. Regarding patients with CSBD of the C^+^subtype we found higher levels of the temperament characteristics *Sensation Seeking* and *Sexual Sensation Seeking* as well as a lower general activation of the *Behavioral Inhibition System*. Patients with alcohol addiction of type II also show higher levels of *Impulsivity* that is conceptually and empirically related to *Sensation Seeking* (80) and low levels of temperament characteristic *Harm Avoidance* (40, 42). It can be concluded that patients with CSBD of the C^+^subtype and patients with alcohol addiction of type II show characteristics that reflect a higher sensitivity to positive reinforcement. However, contrary to patients with CSBD of the C^+^subtype patients with alcohol addiction of type II also show higher levels of temperament characteristic *Novelty-Seeking* (40, 42). As in Cloninger’s theory, the found subtypes of CSBD reflect the two ends of a continuous spectrum of manifestation.

This may have clinical implications for the individual treatment of underlying maintaining mechanisms. Currently, there are only a few studies with randomized controlled trials examining the treatment of CSBD (1). Future studies on the treatment of CSBD should take into account the difference in patients’ sensitivity to reinforcement. Depending on the patient’s position on the continuum, therapy should focus more on the resulting function of the CSBD. Patients of the C^+^subtype use sexual activities to seek reward and experience gratifying effects. Therefore, therapy should focus on building up new and more functional rewarding activities. Since the characteristics of impulsivity and sensation-seeking are conceptually and empirically related (80), and patients of the C^+^subtype are higher in *Sensation Seeking* an additional therapy focus should be on learning impulse control strategies. Therefore, for example, distress tolerance skills of the dialectical behavior therapy (81) can be used. Patients of the Ȼ^−^subtype use sexual activities as a coping strategy and to experience compensatory effects. Therapy should therefore focus more on understanding the individual functionality of sexual activities as coping mechanisms (e.g., to avoid feelings of loneliness or to cope with frustration) and building up, for example, social skills by conducting social skills training or functional coping strategies like acceptance of emotions and emotion regulation strategies to deal with the avoided emotions accordingly, as well as reducing avoidance tendencies.

### 4.1. Limitations

Some limitations need to be kept in mind. Our study is an exploratory analysis of a data collection of patients seeking help at an outpatient psychotherapy clinic. Thus, we had a selective sample without any control sample. Furthermore, our results are restricted to male persons from Western, educated, industrialized, rich, and democratic (WEIRD) countries. Replicating studies with different sexes, sexual orientations, and patients from non-WEIRD countries are needed to prove the generalizability of the results. Additionally, the sample at hand was small. To find a large effect size with a *t*-test we would have needed at least 35 persons per group. Therefore, the small sample size could be one reason for the non-significant difference in anxiety between both clusters. Another limitation is our outdated diagnostic criteria due to the lack of unified official criteria before ICD-11. However, to our knowledge, there is no comparable data set. Additionally, this is the first study that directly addresses differences in types of reinforcement in patients as a susceptibility factor to CSBD. Therefore, there is currently no comparative data. Hence, further research to verify the results using the ICD-11 criteria is needed.

## 5. Conclusion

Our data provided evidence for a two-cluster solution regarding reinforcement sensitivity in patients with CSBD. Patients of cluster 1 showed significantly higher activation of the *Behavioral Inhibition System* and depression, indicating that this cluster represents a C^+^subtype, and patients of cluster 2 showed significantly higher levels of *Sensation Seeking*, *Sexual Sensation Seeking*, and activation of the *Behavioral Approach System* dimension *Fun Seeking* indicating that this cluster represents a Ȼ^−^subtype. In conclusion, there seem to be patients who are more sensitive to positive reinforcement and patients who are more sensitive to negative reinforcement. This may have clinical implications for individual therapy when focusing on underlying maintaining mechanisms.

## Data availability statement

The raw data supporting the conclusions of this article will be made available by the authors, without undue reservation.

## Ethics statement

Ethical approval was not required for the studies involving humans because the study started in 2012. At that time, it was not common in Germany that an ethics application had to be submitted for every study. Instead, the studies were discussed informally in the university outpatient clinic by a committee of experts without the need to formally obtain an ethics vote. The studies were conducted in accordance with the local legislation and institutional requirements. The participants provided their written informed consent to participate in this study.

## Author contributions

SG and RS: conception and design. SG and BW: acquisition of data and analysis and interpretation of data. SG: drafting the article. CM, RS, and BW: revising it for intellectual content. All authors contributed to the article and approved the submitted version.

## References

[1] Antons S Engel J Briken P Krüger THC Brand M Stark R. Treatments and interventions for compulsive sexual behavior disorder with a focus on problematic pornography use: a preregistered systematic review. J Behav Addict. (2022) 11:643–66. doi: 10.1556/2006.2022.00061, PMID: 36083776 PMC9872540

[2] Gola M Lewczuk K Potenza MN Kingston DA Grubbs JB Stark R. What should be included in the criteria for compulsive sexual behavior disorder? J Behav Addict. (2020) 11:160–5. doi: 10.1556/2006.2020.00090, PMID: 34329192 PMC9295236

[3] Harper C Hodgins DC. Examining correlates of problematic internet pornography use among university students. J Behav Addict. (2016) 5:179–91. doi: 10.1556/2006.5.2016.022 27156383 PMC5387769

[4] Kowalewska E Gola M Kraus SW Lew-Starowicz M. Spotlight on compulsive sexual behavior disorder: a systematic review of research on women. Neuropsychiatr Dis Treat. (2020) 16:2025–43. doi: 10.2147/NDT.S221540, PMID: 32943868 PMC7478918

[5] Kraus SW Voon V Potenza MN. Should compulsive sexual behavior be considered an addiction? Addiction. (2016) 111:2097–106. doi: 10.1111/add.13297, PMID: 26893127 PMC4990495

[6] Lew-Starowicz M Coleman E. Mental and sexual health perspectives of the international classification of diseases (ICD-11) compulsive sexual behavior disorder. Commentary to the debate: “Behavioral addictions in the ICD-11. J Behav Addict. (2022) 11:226–9. doi: 10.1556/2006.2022.00032, PMID: 35895453 PMC9295232

[7] Sassover E Weinstein A. Should compulsive sexual behavior (CSB) be considered as a behavioral addiction? A debate paper presenting the opposing view. J Behav Addict. (2022) 11:166–79. doi: 10.1556/2006.2020.00055, PMID: 32997646 PMC9295215

[8] Stark R Klucken T Potenza MN Brand M Strahler J. A current understanding of the behavioral neuroscience of compulsive sexual behavior disorder and problematic pornography use. Curr Behav Neurosci Rep. (2018) 5:218–31. doi: 10.1007/s40473-018-0162-9

[9] Kafka MP. Hypersexual disorder: a proposed diagnosis for DSM-V. Arch Sex Behav. (2010) 39:377–400. doi: 10.1007/s10508-009-9574-7, PMID: 19937105

[10] Karila L Wery A Weinstein A Cottencin O Petit A Reynaud M. Sexual addiction or hypersexual disorder: different terms for the same problem? A review of the literature. Curr Pharm Des. (2014) 20:4012–20. doi: 10.2174/13816128113199990619 24001295

[11] Wordecha M Wilk M Kowalewska E Skorko M Łapiński A Gola M. “Pornographic binges” as a key characteristic of males seeking treatment for compulsive sexual behaviors: qualitative and quantitative 10-week-long diary assessment. J Behav Addict. (2018) 7:433–44. doi: 10.1556/2006.7.2018.33, PMID: 29865868 PMC6174597

[12] Reid RC Carpenter BN Hook JN Garos S Manning JC Gilliland R. Report of findings in a DSM-5 field trial for hypersexual disorder. J Sex Med. (2012) 9:2868–77. doi: 10.1111/j.1743-6109.2012.02936.x, PMID: 23035810

[13] Bőthe B Potenza MN Griffiths MD Kraus SW Klein V Fuss J. The development of the compulsive sexual behavior disorder scale (CSBD-19): an ICD-11 based screening measure across three languages. J Behav Addict. (2020) 9:247–58. doi: 10.1556/2006.2020.00034, PMID: 32609629 PMC8939427

[14] Cooper A Griffin-Shelley E Delmonico DL Mathy RM. Online sexual problems: assessment and predictive variables. Sex Addict Compuls. (2001) 8:267–85. doi: 10.1080/107201601753459964

[15] Cooper A Morahan-Martin J Mathy RM Maheu M. Toward an increased understanding of user demographics in online sexual activities. J Sex Marital Ther. (2002) 28:105–29. doi: 10.1080/00926230252851861 11894795

[16] Fuss J Briken P Stein DJ Lochner C. Compulsive sexual behavior disorder in obsessive-compulsive disorder: prevalence and associated comorbidity. J Behav Addict. (2019) 8:242–8. doi: 10.1556/2006.8.2019.23, PMID: 31079471 PMC7044559

[17] Klein V Rettenberger M Briken P. Self-reported indicators of hypersexuality and its correlates in a female online sample. J Sex Med. (2014) 11:1974–81. doi: 10.1111/jsm.12602, PMID: 24909396

[18] Långström N Hanson RK. High rates of sexual behavior in the general population: correlates and predictors. Arch Sex Behav. (2006) 35:37–52. doi: 10.1007/s10508-006-8993-y, PMID: 16502152

[19] MacLaren VV Best LA. Multiple addictive behaviors in young adults: student norms for the shorter PROMIS questionnaire. Addict Behav. (2010) 35:252–5. doi: 10.1016/j.addbeh.2009.09.023, PMID: 19879058

[20] Odlaug BL Lust K Schreiber LRN Christenson G Derbyshire K Harvanko A. Compulsive sexual behavior in young adults. Ann Clin Psychiatry. (2013) 25:193–200. PMID: 23926574

[21] Dilling H. Freyberger H. J. (2019). Taschenführer zur ICD-10-Klassifikation psychischer Störungen: mit Glossar und diagnostischen Kriterien sowie Referenztabellen ICD-10 vs. ICD-9 und ICD-10 vs. DSM-IV-TR 9. Hogrefe.

[22] Seegers JA. The prevalence of sexual addiction symptoms on the college campus. Sex Addict Compuls. (2003) 10:247–58. doi: 10.1080/713775413

[23] World Health Organization. (2019). Available at: International classification of diseases for mortality and morbidity statistics (11th revision). Available at: https://icd.who.int/browse11/l-m/en

[24] Borgogna NC Aita SL. Another failure of the latent disease model? The case of compulsive sexual behavior disorder. Commentary to the debate: “Behavioral addictions in the ICD-11. J Behav Addict. (2022) 11:615–9. doi: 10.1556/2006.2022.00069, PMID: 36112489 PMC9872533

[25] Brand M Rumpf HJ Demetrovics Z Müller A Stark R King DL. Which conditions should be considered as disorders in the international classification of diseases (ICD-11) designation of “other specified disorders due to addictive behaviors”? J Behav Addict. (2022) 11:150–9. doi: 10.1556/2006.2020.00035, PMID: 32634114 PMC9295220

[26] Briken P Turner D. What does “sexual” mean in compulsive sexual behavior disorder? Commentary to the debate: “Behavioral addictions in the ICD-11.”. J Behav Addict. (2022) 11:222–5. doi: 10.1556/2006.2022.00026, PMID: 35895459 PMC9295231

[27] Efrati Y Kraus SW Kaplan G. Common features in compulsive sexual behavior, substance use disorders, personality, temperament, and attachment. Int J Environ Res Public Health. (2021) 19:296. doi: 10.3390/ijerph19010296, PMID: 35010552 PMC8751077

[28] Allen A Kannis-Dymand L Katsikitis M. Problematic internet pornography use: the role of craving, desire thinking, and metacognition. Addict Behav. (2017) 70:65–71. doi: 10.1016/j.addbeh.2017.02.001, PMID: 28214738

[29] Caselli G Ferla M Mezzaluna C Rovetto F Spada MM. Desire thinking across the continuum of drinking behaviour. Eur Addict Res. (2012) 18:64–9. doi: 10.1159/000333601, PMID: 22178806

[30] Field M Munafò MR Franken IHA. A meta-analytic investigation of the relationship between attentional bias and subjective craving in substance abuse. Psychol Bull. (2009) 135:589–607. doi: 10.1037/a0015843 19586163 PMC2999821

[31] Snagowski J Laier C Duka T Brand M. Subjective craving for pornography and associative learning predict tendencies towards cybersex addiction in a sample of regular cybersex users. Sex Addict Compuls. (2016) 23:342–60. doi: 10.1080/10720162.2016.1151390

[32] Mechelmans DJ Irvine M Banca P Porter L Mitchell S Mole TB. Enhanced attentional bias towards sexually explicit cues in individuals with and without compulsive sexual behaviours. PLoS One. (2014) 9:e105476. doi: 10.1371/JOURNAL.PONE.0105476.10.1371/JOURNAL.PONE.0105476, PMID: 25153083 PMC4143289

[33] Carter BL Tiffany ST. Meta-analysis of cue-reactivity in addiction research. Addiction. (1999) 94:327–40. doi: 10.1046/j.1360-0443.1999.9433273.x 10605857

[34] Klucken T Wehrum-Osinsky S Schweckendiek J Kruse O Stark R. Altered appetitive conditioning and neural connectivity in subjects with compulsive sexual behavior. J Sex Med. (2016) 13:627–36. doi: 10.1016/j.jsxm.2016.01.013, PMID: 26936075

[35] Love T Laier C Brand M Hatch L Hajela R. Neuroscience of internet pornography addiction: a review and update. Behav Sci. (2015) 5:388–433. doi: 10.3390/bs5030388, PMID: 26393658 PMC4600144

[36] Draps M Sescousse G Potenza MN Marchewka A Duda A Lew-Starowicz M. Gray matter volume differences in impulse control and addictive disorders: an evidence from a sample of heterosexual males. J Sex Med. (2020) 17:1761–9. doi: 10.1016/J.JSXM.2020.05.007, PMID: 32690426

[37] Brand M Wegmann E Stark R Müller A Wölfling K Robbins TW. The interaction of person-affect-cognition-execution (I-PACE) model for addictive behaviors: update, generalization to addictive behaviors beyond internet-use disorders, and specification of the process character of addictive behaviors. Neurosci Biobehav Rev. (2019) 104:1–10. doi: 10.1016/j.neubiorev.2019.06.032, PMID: 31247240

[38] Brand M Young KS Laier C Wölfling K Potenza MN. Integrating psychological and neurobiological considerations regarding the development and maintenance of specific internet-use disorders: an interaction of person-affect-cognition-execution (I-PACE) model. Neurosci Biobehav Rev. (2016) 71:252–66. doi: 10.1016/j.neubiorev.2016.08.033, PMID: 27590829

[39] Barker TV Buzzell GA Fox NA. Approach, avoidance, and the detection of conflict in the development of behavioral inhibition. New Ideas Psychol. (2019) 53:2–12. doi: 10.1016/j.newideapsych.2018.07.001, PMID: 31105378 PMC6518416

[40] Cloninger CR. Neurogenetic adaptive mechanisms in alcoholism. Science. (1987) 236:410–6. doi: 10.1126/science.2882604, PMID: 2882604

[41] Cloninger CR Bohman M Sigvardsson S. Inheritance of alcohol abuse. Arch Gen Psychiatry. (1981) 38:861–8. doi: 10.1001/archpsyc.1981.01780330019001, PMID: 7259422

[42] Cloninger CR Sigvardsson S Bohman M. Childhood personality predicts alcohol abuse in young adults. Alcohol Clin Exp Res. (1988) 12:494–505. doi: 10.1111/j.1530-0277.1988.tb00232.x 3056070

[43] Cloninger CR Sigvardsson S Bohman M. Type I and type II alcoholism: an update. Alcohol Health Res World. (1996) 20:18–23. PMID: 31798167 PMC6876531

[44] Cloninger CR. Temperament and personality. Curr Opin Neurobiol. (1994) 4:266–73. doi: 10.1016/0959-4388(94)90083-3 8038587

[45] Cloninger CR. A systematic method for clinical description and classification of personality variants. Arch Gen Psychiatry. (1987) 44:573. doi: 10.1001/archpsyc.1987.01800180093014 3579504

[46] Gray JA. Perspectives on anxiety and impulsivity: a commentary. J Res Pers. (1987) 21:493–509. doi: 10.1016/0092-6566(87)90036-5

[47] Gray JA. Brain systems that mediate both emotion and cognition. Cognit Emot. (1990) 4:269–88. doi: 10.1080/02699939008410799

[48] Gray JA McNaughton N In: Mackintosh NJ Shallice T Treisman A McGaugh JL Schacter D Weiskrantz L, editors. The neuropsychology of anxiety: An enquiry into the functions of the septo-hippocampal system. 2nd ed: New York: Oxford University Press (2000).

[49] Kühn S Gallinat J. A quantitative meta-analysis on cue-induced male sexual arousal. J Sex Med. (2011) 8:2269–75. doi: 10.1111/J.1743-6109.2011.02322.X, PMID: 21599838

[50] Markert C Klein S Strahler J Kruse O Stark R. Sexual incentive delay in the scanner: sexual cue and reward processing, and links to problematic porn consumption and sexual motivation. J Behav Addict. (2021) 10:65–76. doi: 10.1556/2006.2021.00018, PMID: 33822748 PMC8969854

[51] Stoléru S Fonteille V Cornélis C Joyal C Moulier V. Functional neuroimaging studies of sexual arousal and orgasm in healthy men and women: a review and meta-analysis. Neurosci Biobehav Rev. (2012) 36:1481–509. doi: 10.1016/J.NEUBIOREV.2012.03.006, PMID: 22465619

[52] Wehrum-Osinsky S Klucken T Kagerer S Walter B Hermann A Stark R. At the second glance: stability of neural responses toward visual sexual stimuli. J Sex Med. (2014) 11:2720–37. doi: 10.1111/jsm.12653, PMID: 25117824

[53] Voon V Mole TB Banca P Porter L Morris L Mitchell S. Neural correlates of sexual cue reactivity in individuals with and without compulsive sexual behaviours. PLoS One. (2014) 9:e102419. doi: 10.1371/journal.pone.0102419, PMID: 25013940 PMC4094516

[54] Hofmann W Friese M Gschwendner T. Men on the “pull.”. Soc Psychol. (2009) 40:73–8. doi: 10.1027/1864-9335.40.2.73

[55] Kahveci S van Bockstaele B Blechert J Wiers RW. Pulling for pleasure? Erotic approach-bias associated with porn use, not problems. Learn Motiv. (2020) 72:101656. doi: 10.1016/j.lmot.2020.101656

[56] Costumero V Barrós-Loscertales A Bustamante JC Ventura-Campos N Fuentes P Rosell-Negre P. Reward sensitivity is associated with brain activity during erotic stimulus processing. PLoS One. (2013) 8:e66940. doi: 10.1371/journal.pone.0066940, PMID: 23840558 PMC3695981

[57] Snagowski J Brand M. Symptoms of cybersex addiction can be linked to both approaching and avoiding pornographic stimuli: results from an analog sample of regular cybersex users. Front Psychol. (2015) 6:139606. doi: 10.3389/FPSYG.2015.00653/BIBTEX PMC4441125 26052292

[58] Sklenarik S Potenza MN Gola M Astur RS. Approach bias for erotic stimuli among heterosexual female college students who use pornography. Addict Behav. (2020) 108:106438. doi: 10.1016/J.ADDBEH.2020.106438 32325387

[59] Sklenarik S Potenza MN Gola M Kor A Kraus SW Astur RS. Approach bias for erotic stimuli in heterosexual male college students who use pornography. J Behav Addict. (2019) 8:234–41. doi: 10.1556/2006.8.2019.31, PMID: 31257916 PMC7044553

[60] Efrati Y Gola M. The effect of early life trauma on compulsive sexual behavior among members of a 12-step group. J Sex Med. (2019) 16:803–11. doi: 10.1016/j.jsxm.2019.03.272, PMID: 31080103

[61] Kalichman SC Rompa D. Sexual sensation seeking and sexual compulsivity scales: reliability, validity, and predicting HIV risk behavior. J Pers Assess. (1995) 65:586–601. doi: 10.1207/s15327752jpa6503_16, PMID: 8609589

[62] Rettenberger M Klein V Briken P. The relationship between hypersexual behavior, sexual excitation, sexual inhibition, and personality traits. Arch Sex Behav. (2016) 45:219–33. doi: 10.1007/s10508-014-0399-7, PMID: 25559323

[63] Zuckerman M. Sensation seeking: beyond the optimal level of arousal In: Hillsdale, NJ, editor. Personality and individual differences. New York: L. Erlbaum Associates (1979).

[64] Bancroft J Vukadinovic Z. Sexual addiction, sexual compulsivity, sexual impulsivity, or what: toward a theoretical model. J Sex Res. (2004) 41:225–34. doi: 10.1080/00224490409552230 15497051

[65] Carvalho JP Hopko DR. Behavioral theory of depression: reinforcement as a mediating variable between avoidance and depression. J Behav Ther Exp Psychiatry. (2011) 42:154–62. doi: 10.1016/J.JBTEP.2010.10.001, PMID: 21315876

[66] Moulds ML Kandris E Starr S Wong ACM. The relationship between rumination, avoidance and depression in a non-clinical sample. Behav Res Ther. (2007) 45:251–61. doi: 10.1016/J.BRAT.2006.03.003, PMID: 16631110

[67] Salters-Pedneault K Tull MT Roemer L. The role of avoidance of emotional material in the anxiety disorders. Appl Prev Psychol. (2004) 11:95–114. doi: 10.1016/J.APPSY.2004.09.001

[68] Gray JA. The neuropsychology of anxiety. Issues Ment Health Nurs. (1985) 7:201–28. doi: 10.3109/01612848509009455 2869008

[69] Sun J Luo Y Chang H Zhang R Liu R Jiang Y. The mediating role of cognitive emotion regulation in BIS/BAS sensitivities, depression, and anxiety among community-dwelling older adults in China. Psychol Res Behav Manag. (2020) 13:939–48. doi: 10.2147/PRBM.S269874, PMID: 33204187 PMC7667197

[70] Carver CS White TL. Behavioral inhibition, behavioral activation, and affective responses to impending reward and punishment: the BIS/BAS scale. J Pers Soc Psychol. (1994) 67:319–33. doi: 10.1037/0022-3514.67.2.319

[71] Laux L Glanzmann P Schaffner P Spielberger CD. Das State-Trait-Angstinventar (STAI): theoretische Grundlagen und Handanweisung. 1st ed Beltz Test GmbH (1981) https://fis.uni-bamberg.de/handle/uniba/26756.

[72] Hautzinger M Keller F Kühner C. Beck depressions-Inventar (BDI-II). Revision. In: Deutsche Bearbeitung von Beck AT Steer RA Brown GK, editors. Frankfurt/Main: Harcourt test services. 2nd ed: San Antonio, TX: Harcourt Assessment Inc. (2006).

[73] Beauducel A Strobel A Brocke B. Psychometrische Eigenschaften und Normen einer deutschsprachigen Fassung der Sensation Seeking-Skalen. Form V Diagnostica. (2003) 49:61–72. doi: 10.1026//0012-1924.49.2.61

[74] Hammelstein P. Die deutschsprachige Version der Sexual Sensation Seeking Scale und der Sexual Compulsivity Scale. Zeitschrift Für Sexualforschung. (2005) 18:135–47. doi: 10.1055/s-2005-836652

[75] Stark R Kagerer S Walter B Vaitl D Klucken T Wehrum-Osinsky S. Trait sexual motivation questionnaire: Concept and validation. J Sex Med. (2015) 12:1080–91. doi: 10.1111/jsm.12843, PMID: 25676099

[76] R Core Team. (2022). R: A language and environment for statistical computing. R Foundation for Statistical Computing. Available at: https://www.R-project.org/

[77] Tibshirani R Walther G Hastie T. Estimating the number of clusters in a data set via the gap statistic. J Royal Stat Soc Series B Stat Methodol. (2001) 63:411–23. doi: 10.1111/1467-9868.00293

[78] Schubert E Rousseeuw PJ. Faster k-medoids clustering: improving the PAM, CLARA, and CLARANS algorithms In: Amato G Gennaro C Oria V Radovanović M, editors. Lecture notes in computer science (including subseries lecture notes in artificial intelligence and lecture notes in bioinformatics), 11807 LNCS (2019). 171–87.

[79] Mathew RJ Weinman ML. Sexual dysfunctions in depression. Arch Sex Behav. (1982) 11:323–8. doi: 10.1007/BF01541593 7149967

[80] Zuckerman M. Sensation seeking and impulsivity: a marriage of traits made in biology? In: McCown WG Johnson JL Shure MB, editors. The impulsive client: Theory, research, and treatment. Washington, DC: American Psychological Association (1993). 71–91. doi: 10.1037/10500-000

[81] Linehan M. M. (1993). Skills training manual for treating borderline personality disorder. The Guilford Press. Available at: https://psycnet.apa.org/record/1995-98090-000

